# Chemorheology of a Si/Al > 3 Alkali Activated Metakaolin Paste through Parallel Differential Scanning Calorimetry (DSC) and Dynamic Mechanical Analysis (DMA)

**DOI:** 10.3390/polym15193922

**Published:** 2023-09-28

**Authors:** Raffaella Aversa, Laura Ricciotti, Valeria Perrotta, Antonio Apicella

**Affiliations:** Advanced Materials Laboratory, Department of Architecture and Industrial Design, University of Campania, Via San Lorenzo, 81031 Aversa, Italy; laura.ricciotti@unicampania.it (L.R.); valeria.perrotta@unicampania.it (V.P.)

**Keywords:** chemorheology, geopolymer, viscoelasticity, metakaolin, 3dprinting, additive manufacturing, mechanical analysis, differential scanning calorimetry, thermal analysis

## Abstract

Although geopolymers, as structural materials, should have superior engineering properties than traditional cementitious materials, they often need to improve their final characteristics’ reproducibility due to the need for more control of the complex silico-aluminate decomposition and polymerisation stages. Thermosetting of a reactive geopolymeric paste involves tetrahedral Silicate and Aluminate precursor condensation into polyfunctional oligomers of progressively higher molecular weight, transforming the initial liquid into a gel and a structural solid. Viscosity and gelation control become particularly critical when the geopolymer is processed with 3D printing additive technology. Its physical state modification kinetics should match the flow and setting characteristics required by the deposition process. The reaction kinetics and the elastic and viscous characteristics preceding gelation and hardening have been investigated for an alkali-activated Metakaolin/Sodium Silicate-Sodium Hydroxide paste with a Si/Al ratio > 3. A chemoreological approach has been extended to these inorganic polymerisable systems, as already utilised for organic thermosetting polymers. Differential scanning calorimetry and Oscillatory DMA were carried out to monitor the advancement of the polymerisation reaction and the associated variations of the rheological viscoelastic properties. Dynamic thermal scans were run at 1 °C/min and a frequency of 10 Hz for the dynamic mechanical tests. The observed kinetics of polymerisation and variations of the elastic and viscous components of the complex viscosities and shear moduli are described in terms of polycondensation of linear and branched chains of oligomeric macromolecules of increasing complexity and molecular weight up to gelation (Gel1) and cross-linking of the gelled macrostructure (Gel2) and final glassy state. Geopolymerization can be allocated into two main behavioural zones: a viscoelastic liquid paste below 32.5% of reaction advancement and a viscoelastic solid above. Initial complex viscosities range from 2.3 ± 0.9  × 10^−5^ MPa_*_s to 6.8 ± 0.9  × 10^−2^ in the liquid-like state and from 1.9 ± 0.1 MPa to 9.6 ± 2.1 × 10^2^ MPa in the solid-like state.

## 1. Introduction

Geopolymer is the general term encompassing multiphased ceramic materials formed by recombining dissolved species generated by alkali attacks on solid aluminosilicates. An amorphous 3D aluminosilicate network can develop in the alkaline-activated slurry by polycondensation of the tetrahedrally coordinated silicate and aluminate ions [1]. Cement based on these inorganic polymers is becoming a central key point in the development of greener technologies in the heavily polluting building industries [2,3] either for their superior mechanical properties or because of the utilisation of waste and raw materials requiring lower amounts of energy for their production [4,5,6].

Currently, additive technology is becoming more and more popular in different areas, including its applications in the construction industry [7,8,9,10]. However, the growing interest of the construction industry in greener technologies was not only addressed to the development and use of new geopolymeric materials but also to the application of new additive manufacturing technologies that can ensure direct 3D printing of the cementitious slurry into its final structure without casting it into temporary formworks. The potential for high productivity drove such a strategic choice. Additive manufacturing technology reduces environmental and production costs since it can reduce material impact, construction time, and the number of workers while ensuring safer work conditions, the use of waste materials, and new architectural opportunities [4,5,6,7,8,9,10].

The strong interest in understanding the science and technology relationships behind the geopolymerization chemo-physical properties, especially in their use in new additive manufacturing technologies, is responsible for the flourishing scientific studies to improve their properties and reliability [11,12,13,14,15,16,17].

Geopolymerization is an exothermic reaction resulting in the formation of compact, amorphous, or semi-crystalline solid materials. The developing macromolecular structure consists of alternately interlinked alumina and silica tetrahedra sharing oxygen atoms. Therefore, a geopolymer behaves as an inorganic polymer linked by covalent bonds that have a –[Si–O]_n_–Al–O– backbone [7,8,9,10] where “n” depends on the Si/Al ratio in the geopolymer binders and activating solution slurry [18]. Due to the higher Si–O–Si bond strengths compared to those of Si–O–Al and Al–O–Al ones [19,20], the strength of these materials can be modulated accordingly by increasing the silica content of the reacting paste. Alkali metal salts and hydroxide are necessary to dissolve the silico-aluminate and catalyse the polycondensation reactions [19]. Due to the negative valence of the tetrahedral Aluminium-oxygen coordination, dissolved alkali metal cations must be present to keep the macromolecule structure neutral.

Considering that the synthesis and processing of geopolymers and traditional concretes are two different processes [1,9], a completely different methodological approach is needed.

The cure of an alkali-activated geopolymer implicates the transformation of low-molecular-weight monomeric units or oligomers from a liquid to a solid state due to the formation of macromolecules with progressively higher molecular weight and crosslinked polymeric networks. However, these inorganic polymers, which as solid materials could potentially have superior engineering properties than traditional concrete, often suffer in reaching the expected properties because of poor control during the hard setting process [8,9]. Viscosity and gelation properties during processing may vary with temperature, flow condition, and time, according to the kinetics of the chemical reactions occurring in the liquid state. Due to their intrinsic amorphous or semicrystalline nature, precise structural models could not be proposed for geopolymers. Nonetheless, spectroscopic methods and density functional theory computational codes have been used to investigate the structural characteristics of complex geopolymeric macromolecules combining small oligomeric species in 3D networks [21,22].

Due to the complexity of the concurrent gel chemistry and rheology, mathematical modelling and complete chemorheological characterisations become necessary if we want these materials to develop their full technical and commercial potential [9].

Chemorheology, first introduced in ‘1980 to model and design organic polymer composite autoclave processing [23,24], correlates the viscosity variation with the chemical reactions and represents the scientific discipline studying the viscoelastic behaviour of a reacting system [23,24,25,26,27,28,29].

We intend to apply this scientific approach to the processing and additive manufacturing technologies of these inorganic polymers. The viscosity—time profiles of a 3D extruded still plastic geopolymeric cementitious paste must make possible its simultaneous deposition before turning into gel ceasing to flow and the ability to self-sustain its shape once deposited. These flow and structural properties depend on the evolution of the relative viscous and elastic components during the geopolymeric paste transformation from liquid to solid.

The novelty of the proposed experimental approach based on the parallel characterisation of the polymerisation kinetics and viscoelastic behaviour of a geopolymeric paste opens up new studies to develop theoretical or phenomenological models for geopolymer processing. As already approached by the Authors for organic polymer multiphase systems [23,24,25], these models could be used to define the appropriate temperature-time programming of their processing in 3D printing processes.

## 2. Materials and Methods

### 2.1. Materials and Sample Preparation Procedures

The compositions of the high-purity Metakaolin (ASTM C-618 [29] Class N pozzolans) (MetaMax^®^ BASF, NewYork, NY, USA) and Sodium silicate solution (Prochin Italia S.r.l., Caserta, Italy) used to prepare the alkali-activated reacting paste are reported in Table 1. Reagent-grade Sodium hydroxide anhydrous pellets (Sigma-Aldrich, St. Louis, MO, USA) were used to prepare the alkaline activating solution according to the procedures described in a previous paper [30].

The alkaline activating solution, prepared by dissolving Sodium hydroxide in the Sodium silicate solution in a 1/7.7 weight ratio, was equilibrated at room temperature for 24 h before adding the solid Metakaolin [30,31]. Metakaolin solid powder was incorporated into the liquid alkaline activating solution maintained at 5 °C in a thermostated bath in a liquid-to-solid ratio of 1/1.7, mechanically mixed, and sonicated for 10 min.

The final composition of the whole geopolymeric system was previously determined by EDS analysis in a previous paper [30] as Al_2_O_3_ 3.48 SiO_2_ 1.0 Na_2_O 12.14 H_2_O with a molar ratio Silica/Alumina of 3.48.

### 2.2. Thermoanalysis Methods and Testing Procedures

The freshly made reacting paste was tested simultaneously in the differential scanning calorimeter (DSC) and the Dynamical Mechanical Analyser (DMA). The DSC and DMA equipment were controlled by Mettler STAR^e^ Software 18.0 using a multichannel input interface for parallel data exchange. A heating rate of 1 °C/min was utilised for both characterisations.

#### 2.2.1. Differential Scanning Calorimetry

The heat released during the geopolymerization of thermally scanned samples has been monitored using a Mettler ADSC Differential Scanning Calorimeter equipped with a liquid nitrogen cooling unit driven by the Mettler software STAR^e^ 18.0. 

A preliminary isothermal DCS scan on the freshly made slurry was run for twelve hours at 5 °C, followed by a second thermal scan carried out from 5 to 120 °C at 1 °C/min. No reactivity was detected in the isothermal test or in the dynamic thermal scan before 20 °C. All subsequent temperature scans were conducted in a nitrogen atmosphere between 5° and 95 °C at a heating rate of 1 °C/min. For each test, about 30 mg of alkaline-activated metakaolin freshly prepared paste was poured and sealed into a medium-pressure crucible (Mettler MP 120 μL) to avoid water evaporation during the test (this crucible resists pressures up to 2 MPa) and then readily placed in the DSC oven for scanning.

The advancement of the geopolymeric reaction has been expressed in % of the final heat of the reaction (evaluated from the total area under thermograms) and the measured partial area at a determined temperature during the thermal scan. 

The thermocalorimetric scans were replicated on five new, freshly made samples. Statistical analysis was performed on each set of derived data to define their means and standard deviations.

#### 2.2.2. Dynamic Mechanical Analysis Test Procedures and Theoretical Bases

The dynamic-mechanical analyses have been run on a Mettler Toledo Dynamic Mechanical Analyzer (DMA-SDTA 1+) operating in shear force (max 0.5 N) and displacement (max 10 μm) control modes and at a frequency of 10 Hz. 

The oscillatory shearing viscoelastic properties of the alkali-activated metakaolin paste thermally scanned from 25 to 95 °C were continuously monitored from its initial liquid to its final solid state.

**Figure 1:**
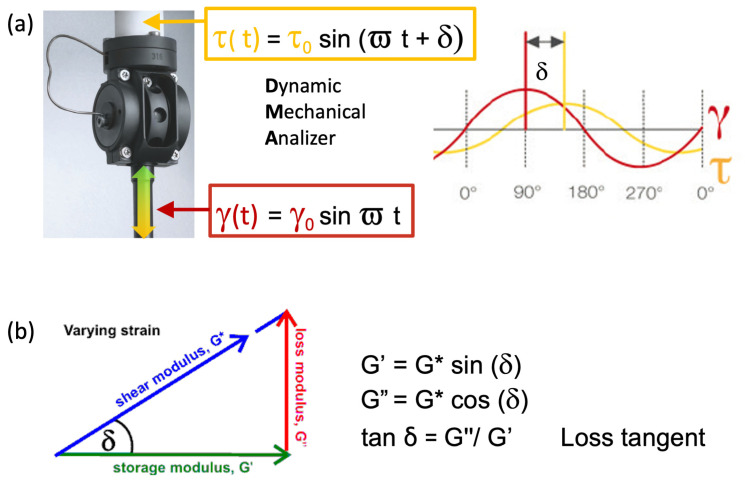
Viscoelasticity analysis tools: (**a**) DMA shear test tool and viscoelastic behaviour measurements; (**b**) Vector representation of complex shear modulus and storage and loss component relationships.

The DMA applies an oscillatory deformation to the material to separate the elastic and viscous components. Two identical weight paste specimens were symmetrically sandwiched in the sample assembly reported in Figure 1a (with a 1.0 to 1.5 mm gap) between three circular steel plates of 10 mm diameter. The two stationary outer parts are connected to a force sensor. At the same time, the central one moves at a controlled sinusoidal strain (see Figure 1a) of controlled amplitude (maximum displacement Δx = 10 μm) and oscillation frequency of 10 Hz.

The upper sensor (the white element in the shear assembly in Figure 1a) detects the counter forces and, hence, the shear stresses τ(t) calculated from the sample geometry needed to keep the side plates in position and needed to extrapolate the apparent shear modulus G* containing both the elastic and viscous responses.

Vectorial representation and complex numbers with a real and an imaginary part may be used to represent variables containing two components [28,32], in our case, a vector on the complex plane diagram shown in Figure 1b:

G* = G′ + *i*G″ (1)

The magnitudes of the elastic (storage modulus) and viscous (loss modulus) vectors are:

G′ = G* sin(δ)           Storage modulus G″= G* cos(δ)             Loss modulus(2)

The phase angle shift δ, which represents the direction of the complex modulus vector, is evaluated from the time lag between the applied sinusoidal oscillation and the resulting shear stress response monitored as a function of the testing time (red and yellow lines, respectively, in Figure 1a).

The direction of the elastic vector (storage modulus), which belongs to the purely elastic behaviour of the tested medium, is parallel to the direction of the applied deformation (the horizontal axis in Figure 1c). Conversely, the direction of the viscous vector (loss modulus) is orthogonal to that of the applied deformation (vertical axis in Figure 1b).

The results of our modulated shear tests may be equivalently represented using shear dynamic viscosities (Eta*, Eta′, and Eta″ in Pa_*_s) as well as shear moduli (G*, G′, and G″ in MPa) according to:

Eta′ = G″/ϖ Eta″ = G′/ϖ(3)

Moreover, data can also be analysed by referring to the ratio between the loss and storage mechanical response components derived from Equations (2) and (3), which is designated as the *loss tangent*. This parameter provides a measure of the relative predominance of the dissipative (tanδ > 1) and elastic (0 < tanδ < 1) characteristics of the material:

tanδ = G″/G′ = Eta′/Eta″(4)

Each thermo-mechanical analysis was replicated on five new, freshly made samples. Statistical analyses were performed on each set of derived data to define their means and standard deviations.

## 3. Results

### 3.1. Parallel Differential Scanning Calorimetry and Dynamic Mechanical Analysis

**Figure 2:**
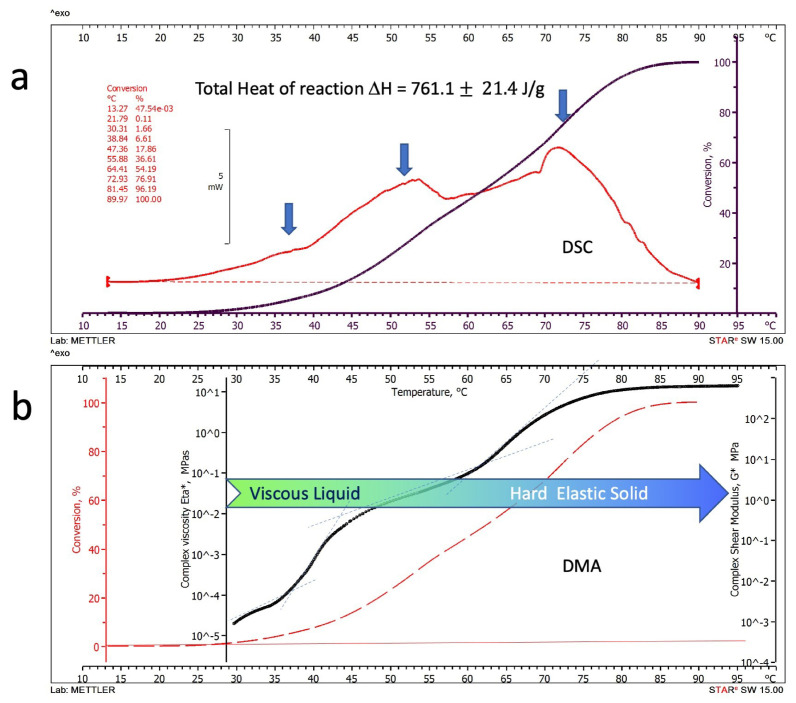
Parallel thermal scan thermograms of the alkaline-activated Metakaolin paste heated at 1 °C/min: (**a**) Differential Scanning Calorimetry thermogram; (**b**) Dynamic Mechanical Analysis thermogram, red dotted line represent the % conversion of the geopolymerization reactions from DSC.

The aim of the parallelly executed thermocalorimetric and thermomechanical tests was to correlate the thermal events exhibited in the DSC thermograms, which depend on the chemical reactions associated with the progression of geopolymerization of the alkaline-activated metakaolin paste, with the physical events detected by the dynamic thermomechanical analysis that can be associated with the increasing macromolecular complexity. According to this methodological approach, Figure 2 reports and compares DSC and DMA thermogram-derived data expressed as heat fluxes (a) and complex viscosities and shear moduli (b), respectively.

#### 3.1.1. Differential Scanning Calorimetry

The heat released during the thermal DSC scan of the Metakaolin alkaline-activated paste heated at 1 °C/min is reported as a red thermogram in Figure 2a as a function of temperature. All five tested specimens showed good reproducibility of the resulting thermograms. The typical thermogram reported in Figure 2a shows three zones of apparently different reactivities, as evidenced by the blue arrows (peaks at 37.2 ± 2.2 °C, 53.6 ± 3.5 °C and 73.2 ± 4.1 °C) indicating that different concurrent reaction mechanisms may be responsible for the observed thermal events. A mean overall heat of the reaction of 761.1 ± 21.4 J/g was calculated from the area under the thermograms of the five tested specimens. The advancement of the reaction expressed as a percentage of the final overall heat of the reaction is reported in Figure 2a as a black line. It can be observed that measurable heat fluxes started at temperatures higher than about 30 °C. Second thermal scans performed on the already-scanned samples showed no residual reactivity up to 120 °C.

#### 3.1.2. Dynamic Mechanical Analysis

The semi-logarithmic diagram of a typical DMA thermogram of a test run on the DSC twinned specimens heated at the same rate is reported in Figure 2b as a function of the scanning temperature. The thermogram (black line) can be equivalently represented, according to Equation (3), as complex viscosity Eta* (left axis in MPa_*_s) or complex shear modulus G* (right axis in MPa). In this paper, although we will maintain both notations and axes in the DMA thermograms, we will preferably discuss the results regarding shear modulus for simplicity.

The reacting paste’s complex viscosities (or equivalently the shear moduli) increase during the polymerisation of about six decades from 10^−5^ to 10 MPa_*_s (10^−3^ to 10^3^ MPa for the complex shear moduli). 

As indicated in Figure 2b, the alkaline-activated metakaolin paste passes from an initially viscoelastic liquid to a final hard elastic solid. At the beginning of the thermal scan at 30 °C, the sample behaves as a liquid with a viscosity of 2.3 ± 0.9  × 10^−5^ MPa_*_s, comparable to that of a thick oil or low-viscosity polymer melt [33]. At the end of the thermal scan at 95 °C, the geopolymer reaches its final solid state with a complex shear modulus of 9.6 ± 2.1 × 10^2^ MPa, comparable to a glassy engineering polymer [33].

Finally, the percentage of advancement of the reaction obtained from the DSC thermogram is also reported as a dotted red line in Figure 2b.

The mechanical properties increase with temperature following the progressive advancement of the chemical reactions up to their exhaustion when the material reaches its final steady value of the complex shear modulus. However, this increase is not monotonically proportional to the conversion but shows abrupt changes in correspondence with the peaks observed on the DSC thermogram. In the range of temperatures between 25 and 35 °C and at a very low conversion level (below 4%), viscosity linearly increases with the temperature. A change in the curve slope is observed above 37 °C. Similar behaviour with changes in the thermogram slope is observed above 44° and 65 °C, corresponding to a chemical conversion of about 12% and 48%. These changes may be associated with modifying geopolymer macromolecular structure, genesis, and hard-setting mechanisms.

### 3.2. Viscoelasticity Investigations

A deeper understanding of the relationships between the chemistry of geopolymerization and macromolecular evolution during the dynamic thermal cure can be obtained by analysing the relative viscous dissipative and elastic storage components and evaluating the relative prevalence of the elastic and viscous behaviour using the loss tangent values.

**Figure 3:**
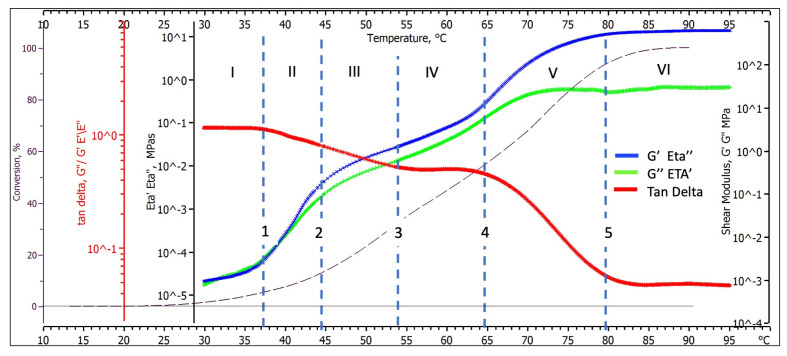
Viscoelastic parameters and reaction kinetics of thermo-scanned at 1 °C/min alkaline-activated Metakaolin paste from DSC and DMA thermograms.

Figure 3 compares the elastic (blue thermogram) and viscous (green thermogram) component variations of the measured complex viscosity Eta* (Eta″ and Eta′, left central logarithmic axis) and, equivalently, shear modulus G* (G′ and G″, logarithmic right axis) as a function of the cure scan temperature. The same figure also reports the loss tangent (tan delta, red logarithmic axis, and red line) and the degree of advancement of the polymerisation (left external axis and black dotted line).

#### 3.2.1. Elastic (Eta″, G′) and Viscous (Eta′, G″) Shear Behaviour during Geo-Polymerisation

As already presented for the complex property, its elastic (blue thermogram) and viscous (green thermogram) components progressively increase as the level of conversion and temperature increase (black dotted line), maintaining the same non-monotonic growth and temperature transitions. However, the viscous component thermogram (green), which overlaps the elastic one (blue) up to 37 °C, progressively diverges to lower values as temperature and conversion progress.

In the early stages of the thermal scan at 30 °C, when the sample is still liquid, the value of the shear modulus viscous component G″ is 1.1 ± 0.3  × 10^−3^ MPa. In comparison, it reaches 4.5 ± 0.6 × 10^1^ MPa in its solid state at the end of the thermal scan (95 °C) with a mechanical property increase of about four decades, while, in the same range, the elastic component increases by nearly six decades (its final value is 9.1 ± 0.7 × 10^2^ MPa_*_s at 95 °C).

Both the elastic and the viscous thermograms shown in Figure 3 present the same characteristic transitions observed for the complex modulus, namely:

- A first thermogram inflexion occurs around 37 °C and has been represented by the dotted line 1;
- A second thermogram inflection follows around 44 °C (dotted line 2).
- A third inflection is observed around 65 °C (dotted line 4);
- Both loss and storage shear moduli finally stabilised their values at temperatures higher than 80 °C (line 5 in Figure 3).

Each transition reflects physical and chemical changes occurring in the reacting paste. Additional information on these changes can be obtained by analysing the corresponding thermogram of the loss tangent.

#### 3.2.2. Loss Tangent (Tan Delta) Behaviour during Geo-Polymerisation

The red thermogram shown in Figure 3 represents the variation of the loss tangent and, as previously indicated, an indication of the relative predominance of the viscous or elastic character of the neo-forming macromolecular material at the different stages of geopolymerization. The loss tangent in the initially reacting paste is near unity to stepwise reduce itself to about 0.50 in an intermediate phase and finally precipitate to 0.04. 

It is evident from its course that abrupt characteristic transitions observed on the DMA thermograms of the reacting material are occurring, namely:

- A first inflexion occurs around 37 °C in correspondence with the change noted for the shear moduli (dotted line 1), where the loss tangent starts to decrease from unity;
- A second inflexion follows around 54 °C (dotted line 3), where the thermogram flattens at a value of about 0.5.
- A third inflexion, in correspondence to that of the shear moduli thermogram, occurs around 65 °C (dotted line 4), where the loss tangent starts again to decrease from its steady value of 0.5.
- The last transition occurs at 80 °C (dotted line 5), where the loss tangent thermogram stabilises to its final value of 0.04.

#### 3.2.3. Chemorheology and Viscoelastic Behavioural Zones

Combining the defined transitions evidenced by the shear moduli and loss tangent thermograms (dotted lines 1–5), we can identify six zones of different viscoelastic behaviour, indicated by Roman numbers in Figure 3, corresponding to the specific values of loss and storage viscoelastic properties, loss tangent, and advancement of the geopolymerization reported in Table 2.

The six transition Zones identifying different viscoelastic behaviours are:

- Zone I—The paste behaves as a viscoelastic liquid (the loss tangent is near unity) that smoothly increases its viscosity as the polycondensation reactions proceed up to a conversion of about 3.9%.
- Zone II—The paste still behaves as a viscoelastic liquid with an increased elastic character (the loss tangent is 0.75). The mechanical properties rapidly increase as chemical conversion rises from 3.9 to 12.8.
- Zone III—The paste behaves as a highly elastic fluid (loss tangent decreases from 0.75 to 0.51). The rate at which the mechanical properties increase as chemical conversion proceeds from 12.4 to 32.5% is slower than for zone II;
- Zone IV—The geopolymer reaches a constant loss tangent of 0.51 while smoothly increasing loss and storage mechanical properties. According to these values, the paste behaves as a solid viscoelastic rubber;
- Zone V—The mechanical property storage component increases at a significantly higher rate than the loss one. Accordingly, the loss tangent rapidly decreases from 0.5 to 0.04, assuming the values characteristic of a rigid glassy polymer when conversions rise from 54.7 to 91.3%;
- Zone VI—The loss tangent (0.04) and modulus components (Table 2) in this zone are characteristic of an essentially elastic solid and remain almost constant up to the test end.

## 4. Discussion

**Figure 4:**
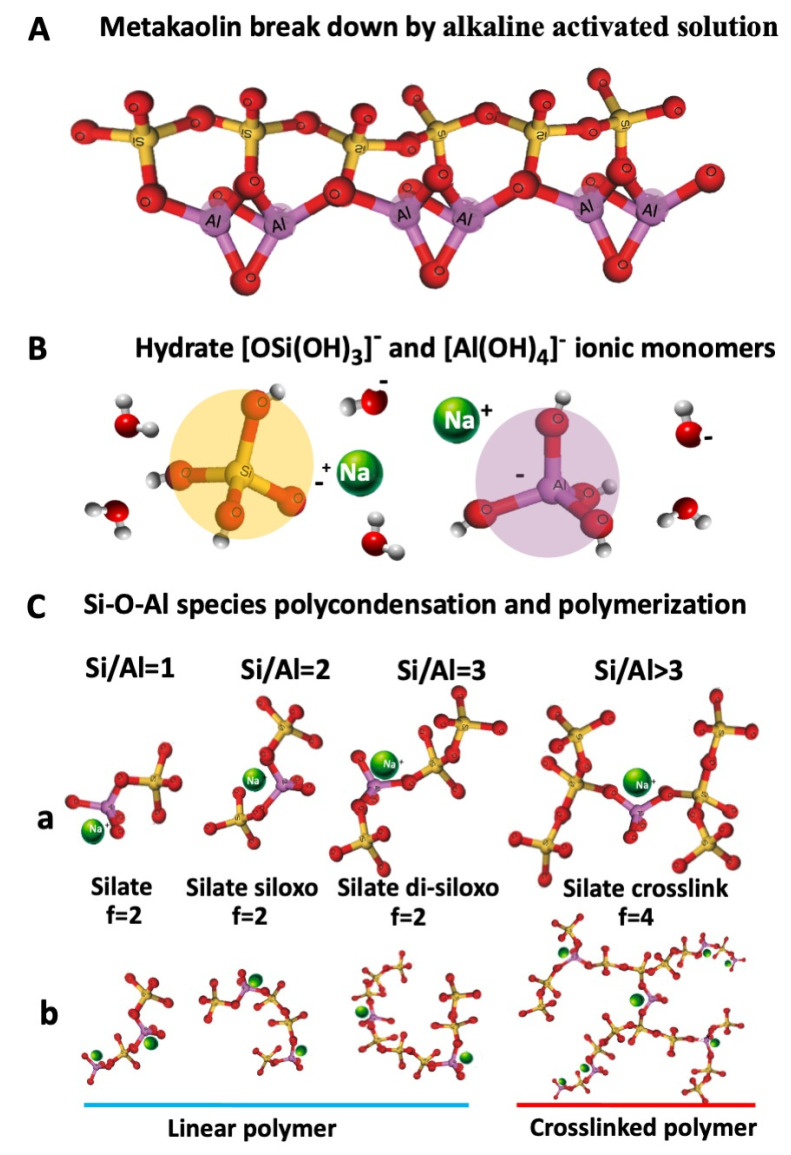
Materials and mechanism of geopolymerization: (**A**) Metakaolin; (**B**) Silicate and Aluminate ionic monomers from kaolin dissolution; (**C**) aluminosilicate molecules (**a**) low Mw oligomers and their functionalities; (**b**) higher Mw linear and branched macromolecules.

Alkaline metal salts and hydroxides (Sodium Silicate and NaOH in our case) are necessary to dissolve the aluminosilicate and to catalyse the polycondensation reaction (Figure 4A). The first silicate and aluminate monomeric units are generated early after adding the alkaline activating solution to the metakaolin precursor. During the alkaline-activated dissolution (Figure 4B), the chemical bonds of the kaolin metastable aluminosilicate are fragmented down into hydrate silicate [OSi(OH)_3_]^−^ and aluminate [Al(OH)**_4_**]^−^ ionic monomers [34,35].

Monomers connect to create dimers as the dissolution progresses, and dimers connect with other monomers to generate trimers, multimers, etc. To keep its structure neutral, sodium cations must be present in the growing molecule. Higher oligomers from polycondensation reactions are formed by joining these monomers. 

Figure 4C shows the low molecular weight (Mw) oligomers that can be formed depending on the relative Si/Al ratio present in the reacting medium [36,37]:

- Silate dimers with a Si/Al = 1
- Silate siloxo trimers with Si/Al = 2
- Silate di-siloxo tetramers with Si/Al = 3
- Branched higher oligomers with Si/Al > 3

As indicated in Figure 4C(a), silate, silate siloxo, and silate di-siloxo form linear polymer chains for Si/Al < 3, behaving then as difunctional (f = 2) oligomeric molecules. For ratios of Si/Al > 3, silate crosslinks generate, leading to tetrafunctional (f = 4) oligomers [37].

In an alkaline environment, the distorted aluminate of the metastable aluminosilicate matrix dissolves more quickly than silicate, leading to a higher concentration of Al during the early stages of the polycondensation reaction [38]. Due to the higher concentration of [Al(OH)**_4_**]^−^, polymeric silate chains with a Si/Al = 1 are statistically preferred and expected to form first.

However, as more [OSi(OH)**_3_**]^−^ groups dissolve, increasing the Si/Al ratio, silate siloxo and silate di-siloxo may form and participate in the formation of higher molecular weight oligomeric chains that can finally cross-link when the Si/Al ratio becomes higher than 3 [38]. This oligomeric solution containing multifunctional elements can branch, forming crosslinked gels [26,38].

The polymerisation of multifunctional monomers is known to pass through the gel transition, a transition that drastically affects the material’s physical properties. The gel point is experimentally well observable due to considerable differences in the material properties between [26] the gel and sol phases, as can occur for viscoelasticity parameters. Hence, it is common practise to define the gel transition point as a point where the observable properties change. Here, we will define the gel transition entirely based on the topological properties of the corresponding storage shear modulus and loss tangent thermograms in Figure 3.

**Figure 5:**
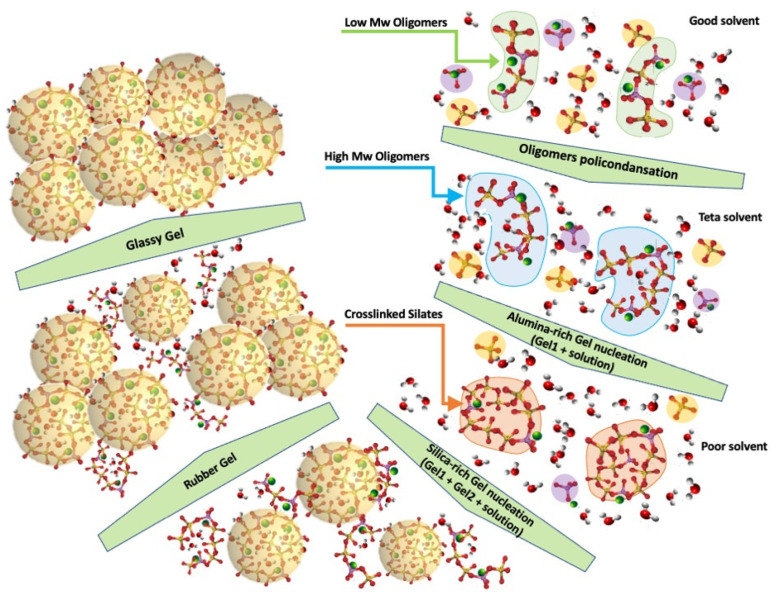
Molecular reassembly mechanism by polycondensation reactions in alkaline-activated geopolymerization.

Figure 5 sketches how the oligomers generated by the metakaolin dissolution could reorganise and polycondense to form an amorphous sodium aluminosilicate hydrate gel with a three-dimensional network structure.

As reactions start and proceed, the low Mw oligomeric chains (right upper side of Figure 5) become progressively restricted compared to the free monomeric groups due to the modification of the solution’s dynamic (rheology) and thermodynamic (solubility) properties [39,40]. 

The interactions of the solvating units (essentially water and the alumina and silica monomeric ionic species) with the growing macromolecule become progressively weaker, reducing its affinity with the solvent. Poor affinity leads to a positive mixing enthalpy [40], and to the polymeric chain’s tendency to bend on itself and segregate from the solution. The random coiled dimension of the neo-forming polymeric macromolecule will progressively decrease as the solution-solvating properties progress from good to teta and poor solvent. This adverse thermodynamic condition favours the nucleation of the first Aluminate-rich “gel” particles (Gel1) containing, as described before, the firstly formed linear silate [41] entrapping solvent molecules and unreacted low molecular weight oligomers [36,37,38,39].

The polycondensation reaction continues in the liquid phase and the gelled particles, creating new small gel particles while increasing the size of the already-formed Gel1 particles (lower right side of Figure 5). 

Under the imposed dynamic thermochemical conditions, kaolin dissolution and polycondensation reactions continue, and more [OSi(OH)_3_]^−^ dissolves, exhausting [Al(OH)_4_]^−^ groups and improving the Si/Al ratio to values higher than 3. Molecular branching and precipitation of Si-rich gel particles (Gel2) are then favoured [41].

A non-regular and charge-balanced aluminosilicate backbone forms by random copolymerisation of linear and branched silate, silate siloxo, and silate di-siloxo, leading to a three-dimensional structure (left lower part of Figure 5). The gel components (Gel1 and Gel2) aggregate into a larger, continuous structure as the polycondensation reaction proceeds. The point at which this event occurs marks the end of a liquid-like state and the transition into solid elastic rubber. Finally, the continuous rubber network formed by the aggregated gel particles continues to crosslink, undergoing vitrification (left upper part of Figure 5). The non-regular copolymeric structure favours the formation of an amorphous glassy geopolymer [42]. Semicrystalline geopolymers have been observed for alkaline-activated aluminosilicates with lower Si/Al ratios [1,42].

**Figure 6:**
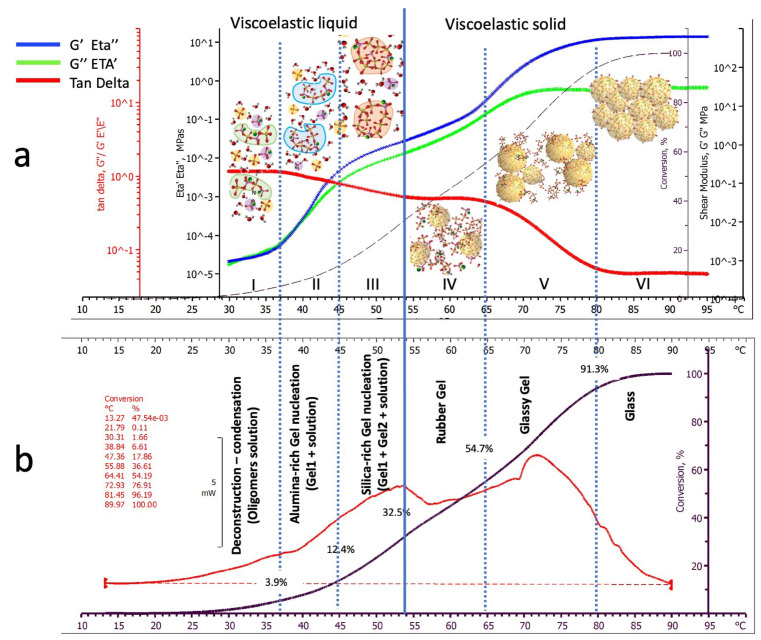
Physical states and viscoelasticity Zones for geopolymerization of an alkaline-activated Metakaolin paste heated at 1 °C/min: (**a**) Dynamic Mechanical Analysis thermograms and hypothesised reacting medium and graphical representation of the morphological composition; (**b**) Characteristic Differential Scanning Calorimetry thermogram with the indication of the conversions corresponding to the transition between viscoelastic behavioural Zones and description of their morphological composition.

Figure 6 summarises the DMA and DSC thermograms and correlates them with the physical states described in Figure 5 and the viscoelastic behavioural Zones defined in Figure 4:

- Zone I: Kaolin deconstruction and silico-aluminate oligomers formation—viscoelastic liquid;
- Zone II: Nucleation of Alumina-rich gel particles—a viscoelastic liquid solution containing Alumina-rich gel particles (Gel1);
- Zone III: Nucleation of Silica-rich gel particles—a viscoelastic liquid solution containing Alumina-rich (Gel1) and silica-rich (Gel2) gel particles;
- Zone IV: Silico-aluminate rubber gel—amorphous viscoelastic rubber;
- Zone V: Silico-aluminate glassy gel (still reactive);
- Zone VI: Silico-aluminate glass geopolymer (fully polymerised)

According to Figure 6, geopolymerization treating can be divided into two main stages: behaving as a viscoelastic liquid or as a viscoelastic solid. The blue vertical full line at 54 °C separates these two behaviours and corresponds to a critical conversion of 32.5% where the material gelation can be located.

The authors have already successfully used the Flory-Stokmayer gelation theory [43,44], first applying percolation process analysis to cross-linking and gelation of step-growth polymerisation, and to epoxy and unsaturated polyester resins containing multifunctional molecules [23,24,25,26,27] to define the conditions for incipient gelation. The critical chemical conversion of the thermosetting reaction (α_c_) is defined as,

α_c_ = 1/(f − 1) (5)

correlating a molecule’s functionality and concentration in a reactive solution containing difunctional molecules able to form linear polymeric chains and molecules with functionality equal to or higher than three able to branch and crosslink [42,43,44,45,46].

According to Equation (8), our observed critical conversion α_c_ = 0.325 corresponds to the functionality of about four, confirming the presence in the reacting system of tetrafunctional molecules as those that can form in our systems at values of Si/Al higher than 3. The alkaline-activated polymerisable mixture then behaves as a processable liquid or a plastic solid up to this level of polymerisation. Beyond this threshold, it cannot be moulded into a new shape. However, although not further processable, the rubber gel can still exhaust its 77.5% reactivity and significantly increase its mechanical characteristics up to vitrification.

## 5. Conclusions

The cure of an alkaline-activated reactive prepolymer involves the transformation of low-molecular-weight monomers or oligomers from a liquid to a rubbery and solid state due to the formation of a polymeric network by the chemical reaction of the reactive groups in the system. Gelation and vitrification, two macroscopic phenomena encountered during this process, strongly alter the viscoelastic behaviour of the material in these early phases of the geopolymerization stages. For long-term property changes, other methods such as variable frequency conductivity, XRD qualitative and quantitative analysis, porosity measurements, and sorption analysis were applied to characterise the process of solidification of geopolymers based on fly ash with sand additives [31,47,48,49,50,51,52].

Gelation, associated with a dramatic viscosity increase, occurs at a critical value of the degree of reaction calculable for each reacting system applying percolation-based theories [31,43,44,45,46,47]. On a molecular level, the viscosity increase corresponds to the rise in molecular weight and the incipient formation of infinitely branched molecules. The growth and branching of the polymer chains occur in the liquid state while the system is still soluble and moldable. In contrast, the infinite network is developed after the gel point by intramolecular reactions of the branched molecules, finally leading to an insoluble elastic crosslinked solid. Vitrification, which usually follows gelation, results from the polymeric network becoming denser through further intramolecular crosslinking that may prevent further reaction by reducing the mobility of the unreacted molecular species or functional groups.

Although chemical control is the basic assumption of all statistical treatments of the cure reactions, in some occurrences, the cure may also be controlled by physical factors such as diffusion constraints in the glass transition region, leading to the formation of no-homogeneous structures that, indirectly, may alter the mechanical properties and durability of the final material.

Knowledge of the structural parameters of the cured systems as a function of the reactivity of the functional species involved in the process and their relative composition is essential in elucidating the curing mechanisms, the control of the process, and material final application properties.

Geopolymer materials should possess many characteristics to meet the critical parameters for 3D printing technology, such as rheological, physical, and mechanical properties, as discussed in the paper and summarised in Figure 6. The key parameters include aluminosilicate raw materials and activator compositions, the eventual presence of reinforcing fillers, geometrical printing variables, curing conditions (temperature and time), and post-processing of the 3D-printed specimens.

The proposed chemorheological approach for the assessment of the more idoneous processing conditions of a specific alkaline-activated aluminosilicate formulation can give insights into the characteristic viscoelastic behavioural Zones of good processability as well as the mechanical and energetical requirements for material extrusion and deposition and their final mechanical characteristics.

Future work will be addressed by evaluating the chemo-rheological behaviour of the same alkaline-activated metakaolin geopolymer under isothermal cure conditions.

## Figures and Tables

**Table 1:** Oxides relative composition ^1^ in Metakaolin and Sodium Silicate Solution raw materials.

Oxide	Metakaolin	Sodium Silicate
**Al_2_O_3_**	45.20	-
**SiO_2_**	52.30	28.36
**K_2_O**	0.15	-
**Fe_2_O_3_**	0.42	-
**Na_2_O**	-	8.60
**MgO**	0.04	
**H_2_O**	-	63.04

^1^ Weight %.

**Table 2:** Viscosity and shear modulus components, temperatures, and reaction conversion ranges identify the chemorheological behaviour of the reacting paste.

ZONE	Temperature, °C	Loss Viscosity, MPa·s	Storage Viscosity, MPa·s	Loss Modulus, MPa	Storage Modulus MPa	Loss Tangent	% of Conversion
I	30.0	2.4 ± 0.4 × 10^−5^	2.3 ± 0.6 × 10^−5^	1.5 ± 0.3 × 10^−3^	1.4 ± 0.2 × 10^−3^	1.1 ± 0.1	0
	37.4	7.7 ± 0.3 × 10^−5^	7.4 ± 0.2 ×10^−5^	4.5 ± 0.2 × 10^−3^	4.6 ± 0.7 × 10^−3^	1.3 ± 0.3	3.9 ± 0.4
II	37.4	-	-	-	-	-	-
	4 4.6	2.3 ± 0.6 × 10^−3^	3.1 ± 0.6 × 10^−3^	1.4 ± 0.4 × 10^−1^	1.8 ± 0.5 × 10^−1^	0.75 ± 0.05	12.4 ± 0.8
III	44.6	-	-	-	-	-	-
	53.9	1.3 ± 0.5 × 10^−2^	2.5 ± 0.4 × 10^−2^	8.2 ± 0.2 × 10^−1^	1.6 ± 0.4 × 10	0.51 ± 0.07	32.5 ± 1.5
IV	53.9	-	-	-	-	-	-
	64.8	8.5 ± 0.8 × 10^−2^	1.9 ± 0.3 × 10^−1^	5.1 ± 0.7	1.2 ± 0.5 × 10	0.43 ± 0.04	54.7 ± 3.9
V	64.8	-	-	-	-	-	-
	79.9	4.6 ± 0.5 × 10^−1^	1.1 ± 0.6 × 10	2.9 ± 0.4 × 10	6.6 ± 0.6 × 10^2^	0.05 ± 0.01	91.3 ± 5.1
VI	79.9	-	-	-	-	-	-
	95.0	5.0 ± 0.4 × 10^−1^	1.3 ± 0.4 × 10	3.2 ± 0.7 × 10	8.2 ± 0.6 × 10^2^	0.04 ± 0.01	100.0

## Data Availability

Not applicable.

## References

[1] Davidovits J. (1991). Geopolymers. J. Therm. Anal..

[2] Mikulčić H., Klemeš J.J., Vujanović M., Urbaniec K., Duić N. (2016). Reducing greenhouse gasses emissions by fostering the deployment of alternative raw materials and energy sources in the cleaner cement manufacturing process. J. Clean. Prod..

[3] The European Cement Industry Association (CEMBUERAU) (2014). The Role of Cement in the 2050 Low Carbon Economy—Full Report.

[4] Zhuang X.Y., Chen L., Komarneni S., Zhou C.H., Tong D.S., Yang H.M., Yu W.H., Wang H. (2016). Fly ash-based geopolymer: Clean production, properties and applications. J. Clean. Prod..

[5] Al-Qutaifi S., Nazari A., Bagheri A. (2018). Mechanical properties of layered geopolymer structures applicable in concrete 3D-printing. Constr. Build. Mater..

[6] Singh N.B. (2018). Fly Ash-Based Geopolymer Binder: A Future Construction Material. Minerals.

[7] Ngo T.D., Kashani A., Imbalzano G., Nguyen K.T., Hui D. (2018). Additive manufacturing (3D printing): A review of materials, methods, applications and challenges. Compos. Part B.

[8] Zhong H., Zhang M. (2022). 3D printing geopolymers: A review. Cem. Concr. Compos..

[9] Provis J.L., Duxson P., Van Deventer J.S.J., Lukey G.C. (2005). The Role of Mathematical Modelling and Gel Chemistry in Advancing Geopolymer Technology. Chem. Eng. Res. Des..

[10] Mierzwiński D., Łach M., Gądek S., Lin W.T., Tran D.H., Korniejenko K. (2023). A brief overview of the use of additive manufacturing of con-create materials in construction. Acta Innov..

[11] Chen Y., Jia L., Liu C. (2022). Mechanical anisotropy evolution of 3D-printed alkali-activated materials with different GGBFS/FA combinations. J. Build. Eng..

[12] Khan S.A., İlcan H., Aminipour E. (2023). Buildability analysis on effect of structural design in 3D concrete printing (3DCP): An experimental and numerical study. Case Stud. Constr. Mater..

[13] Marczyk J., Ziejewska C., Łach M. (2019). Possibilities of using the 3D printing process in the concrete and geopolymers application. IOP Conf. Ser. Mater. Sci. Eng..

[14] Villaquirán-Caicedo M.A., Fernández-González A., Fernández-García D.A., Mejía de Gutiérrez R. (2023). Valorization of a low-quality coal ash, in the preparation of alkali activated inks for applications in 3D additive manufacturing. Constr. Build. Mater..

[15] Duan Z., Deng Q., Liang C., Ma Z., Wu H. (2023). Upcycling of recycled plastic fiber for sustainable cementitious composites: A critical review and new perspective. Cem. Concr. Compos..

[16] Dilawar Riaz R., Usman M., Ali A., Majid U., Faizan M., Jalil Malik U. (2023). Inclusive characterization of 3D printed concrete (3DPC) in additive manufacturing: A detailed review. Constr. Build. Mater..

[17] Aversa R., Petrescu R.V.V., Petrescu F.I.T., Apicella A. (2016). Biomimetic and evolutionary design driven innovation in sustainable products development. Am. J. Eng. Appl. Sci..

[18] Buchwald A., Zellmann H., Kaps C. (2011). Condensation of aluminosilicate gels—Model system for geopolymer binders. J. Non-Cryst. Solids.

[19] Duxson P., Provis J., Lukey G., Mallicoat S., Kriven W., van Deventera J. (2005). Understanding the relationship between geopolymer composition, microstructure and mechanical properties. Colloids Surf. A.

[20] de Jong B.H.W.S., Brown G.E. (1980). Polymerization of silicate and aluminate tetrahedra in glasses, melts, and aqueous solutions: Electronic structure of H6Si2O7, H6AlSiO7, and H6Al2O7. Geochim. Cosmochim. Acta.

[21] Koleżyński A., Król M., Żychowicz M. (2018). The structure of geopolymers. Theoretical studies. J. Mol. Struct..

[22] Walkley B., Rees G.J., San Nicolas R., van Deventer J.S., Hanna J.V., Provis J.L. (2018). New structural model of sodium aluminosilicate gels and the role of charge balancing extra-framework. Al. J. Phys. Chem. C.

[23] Apicella A., Nicolais L., Halpin J.C. Role of the processing chemo-rheology on the ageing behaviour of high performance epoxy matrices. Proceedings of the 28th National SAMPE Symposium and Exhibition.

[24] Halpin J.C., Apicella A., Nicolais L., Astarita G., Nicolais L. (1984). Processing of Thermosets. Polymer Processing and Properties.

[25] Apicella A., Nicolais L., Iannone M., Passerini P. (1984). Thermokinetics and chemorheology of the cure reactions of the tetraglycidyl diamino diphenyl methane–diamino diphenyl sulfone epoxy systems. J. Appl. Polym. Sci..

[26] Apicella A., Pritchard G. (1986). Effect of Chemorheology on Epoxy Resin Properties. Developments in Reinforced Plastics—5.

[27] Nicolais L., Apicella A. (1985). Processing of composite structures. Pure Appl. Chem..

[28] Aversa R., Petrescu R.V.V., Apicella A., Petrescu F.I.T. (2016). One can slow down the aging through antioxidants. Am. J. Eng. Appl. Sci..

[29] (2017). Standard Specification for Coal Fly Ash and Raw or Calcined Natural Pozzolan for Use in Concrete.

[30] Roviello G., Ricciotti L., Ferone C., Colangelo F., Cioffi R., Tarallo O. (2013). Synthesis and Characterization of Novel Epoxy Geopolymer Hybrid Composites. Materials.

[31] Aversa R., Petrescu R.V.V., Petrescu F.I.T., Apicella A. (2016). Smart-factory: Optimization and process control of composite centrifuged pipes. Am. J. Appl. Sci..

[32] Annunziata M., Aversa R., Apicella A., Annunziata A., Apicella D., Buonaiuto C., Guida L. (2006). In vitro biological response to a light-cured composite when used for cementation of composite inlays. Dent. Mater..

[33] Mark J.E. (2006). Physical Properties of Polymers Handbook.

[34] Rifaai Y., Yahia A., Mostafa A., Aggoun S., Kadri E.H. (2019). Rheology of fly ash-based geopolymer: Effect of NaOH concentration. Constr. Build. Mater..

[35] Castillo H., Collado H., Droguett T., Sánchez S., Vesely M., Garrido P., Palma S. (2021). Factors Affecting the Compressive Strength of Geopolymers: A Review. Minerals.

[36] Ng C., Alengaram U.J., Wong L.S., Mo K.H., Jumaat M.Z., Ramesh S. (2018). A review on microstructural study and compressive strength of geopolymer mortar, paste and concrete. Constr. Build. Mater..

[37] Liew Y.-M., Heah C.-Y., Mustafa A.B.M., Kamarudin H. (2016). Structure and properties of clay-based geopolymer cements: A review. Prog. Mater. Sci..

[38] Chen X., Kim E., Suraneni P., Struble L. (2020). Quantitative Correlation between the Degree of Reaction and Compressive Strength of Metakaolin-Based Geopolymers. Materials.

[39] Kryven I., Duivenvoorden J., Hermans J., Iedema P.D. (2016). Random Graph Approach to Multifunctional Molecular Networks. Macromol. Theory Simul..

[40] Flory P.J. (1942). Thermodynamics of high polymer solutions. J. Chem. Phys..

[41] Görhan G., Aslaner R., Şinik O. (2016). The effect of curing on the properties of metakaolin and fly ash-based geopolymer paste. Compos. Part B Eng..

[42] Barbosa V.F., MacKenzie K.J., Thaumaturgo C. (2000). Synthesis and characterization of materials based on inorganic polymers of alumina and silica: Sodium polysialate polymers. Int. J. Inorg. Mater..

[43] Flory P.J. (1941). Molecular Size Distribution in Three Dimensional Polymers I. Gelation. J. Am. Chem. Soc..

[44] Stockmayer W.H. (1944). Theory of Molecular Size Distribution and Gel Formation in Branched Polymers II. General Cross Linking. J. Chem. Phys..

[45] Flory P.J. (1941). Molecular Size Distribution in Three Dimensional Polymers II. Trifunctional Branching Units. J. Am. Chem. Soc..

[46] Flory P.J. (1941). Molecular Size Distribution in Three Dimensional Polymers III. Tetrafunctional Branching Units. J. Am. Chem. Soc..

[47] Sahini M., Sahimi M. (2003). Applications of Percolation Theory.

[48] Kryven I. (2016). Emergence of the giant weak component in directed random graphs with arbitrary degree distributions. Phys. Rev. E.

[49] Mierzwiński D., Walter J., Wanat D. (2023). Possibilities of Checking Water Content in Porous Geopolymer Materials Using Impedance Spectroscopy Methods. Materials.

[50] Walter J., Uthayakumar M., Balamurugan P., Mierzwiński D. (2021). The Variable Frequency Conductivity of Geopolymers during the Long Agieng Period. Materials.

[51] Lolli F., Manzano H., Provis J.L., Bignozzi M.C., Masoero E. (2018). Atomistic Simulations of Geopolymer Models: The Impact of Disorder on Structure and Mechanics. ACS Appl. Mater. Interfaces.

[52] Rubinstein M., Colby R.H. (2003). Polymer Physics.

